# Interpreting rapid diagnostic test (RDT) for *Plasmodium falciparum*

**DOI:** 10.1186/s13104-018-3967-4

**Published:** 2018-12-04

**Authors:** Verner N. Orish, Virtue F. De-Gaulle, Adekunle O. Sanyaolu

**Affiliations:** 1 Department of Microbiology and Immunology, School of Medicine, University of Health and Allied Sciences, Ho, Volta Region Ghana; 2 Department of Social and Behavioural Sciences, School of Public Health, College of Health Sciences, University of Ghana, Accra, P. O. Box LG 13, Legon, Ghana; 3 Department of Medical Microbiology and Parasitology, College of Medicine of the University of Lagos, Idi-araba, Lagos, Nigeria

**Keywords:** Rapid diagnostic tests (RDT), Malaria diagnosis, *Plasmodium falciparum* histidine rich proteins-2 (HRP2), Parasite lactose dehydrogenase enzyme (pLDH)

## Abstract

**Objective:**

Rapid diagnostic tests have been of tremendous help in malaria control in endemic areas, helping in diagnosis and treatment of malaria cases. It is heavily relied upon in many endemic areas where microscopy cannot be obtained. However, caution should be taken in the interpretation of its result in clinical setting due to its limitations and inherent weakness. This paper seeks to present the varying malaria RDT test results, the possible interpretations and explanation of these results common in endemic regions. Published works on malaria RDT studies were identified using the following search terms “malaria RDT in endemic areas”, “*Plasmodium falciparum* and bacterial coinfection” “*Plasmodium falciparum* RDT test results in children in endemic areas” in Google Scholar and PubMed.

**Results:**

The review results show that RDT positive results in febrile patients can either be true or false positive. True positive, representing either a possible single infection of *Plasmodium* or a co-infection of bacteria and *P. falciparum*. False RDT negative results can be seen in febrile patient with *P. falciparum* infection in prozone effect, Histidine rich protein 2 (HRP2) gene deletion and faulty RDT kits. Hence, a scale up of laboratory facilities especially expert microscopy and other diagnostic tools is imperative.

## Introduction

Prompt and accurate malaria diagnosis is important to malaria control programs in endemic regions as it limits over diagnosis as well as provide evidence of infection that requires prompt and adequate treatment [1]. In 2010 for instance, the World Health Organization (WHO) revised the malaria management component of its Integrated Management of Childhood Illness (IMCI) program to an evidence based program of test, treat and track [2]. This is because, the IMCI program formerly recommended that children presenting with fever in malaria endemic regions be treated promptly with anti-malarials [1, 3]. This recommendation was hinged on two main reasons; the first being the limited parasitological diagnostic tools in resource constrained endemic regions and the second being the high prevalence of morbidity and mortality of clinical malaria infections among children in endemic regions [1, 3]. The World Health Organization fully aware of this malaria diagnostic challenge common in endemic regions collaborated with manufactures, scientists and clinicians in the development and introduction into clinical practice a rapid, easy to read and accurate diagnostic test in 2010 [4, 5]. Hence, rapid diagnostic test (RDT) was introduced into clinical management of malaria and since then, more than one million RDTs are used yearly in various health facilities in malaria endemic areas [6, 7]. Although RDT can be used alone in areas where there is no microscopy, it is ideally not meant to replace microscopy which is the gold standard for diagnosis, but rather to complement it [8, 9].

Rapid diagnostic test kits, commonly used in most health facilities in endemic areas include those specific for *Plasmodium falciparum* histidine rich proteins-2 (HRP2) from SD Bioline Malaria Ag Pf^®^ (Standard Diagnostics, Kyonggi, Korea) and First Response Malaria Ag Pf ^®^ (Premier Medical Corporation Ltd, India) [10]. Other less commonly used ones include CareStart Malaria pLDH/HRP2 combo™ (AccessBio Inc., NJ, USA) and Acon Malaria pLDH/HRP2. Comparison studies have shown that there is not much significant difference between HRP2 and pLDH in terms of their effectiveness in detecting malaria parasites [10]. Rapid diagnostic tests employ lateral flow immunochromatographic assay methods in malaria antigens detection. This involves antigen–antibody interactions on a nitrocellulose test strip [11, 12].

Although, malaria RDTs are of tremendous help, a number of studies have highlighted some weaknesses of the test kits. It is therefore important to highlight the varying presentations of RDTs results, and the possible interpretation and explanations. Hence, this paper seeks to present the varied malaria RDT test results common in endemic regions and provide interpretations for these diverse test results by reviewing various published literature on the subject.

## Main text

### Methods

A review of published research work was used to present the diverse malaria RDT test results, provide possible causes and interpretations of the test outcomes. The search words used were “*Plasmodium falciparum* RDT test results in children in endemic areas”, “Malaria RDT in endemic areas”, “*Plasmodium falciparum* and bacterial coinfection” and “Asymptomatic *plasmodium falciparum* in children”. Google scholar and PubMed were the databases used to identify the review documents.

**Fig. 1:**
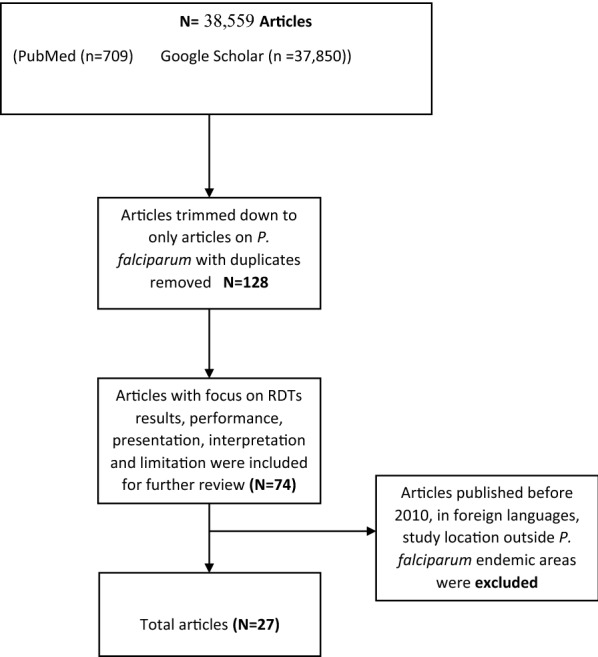
Flow diagram of the article search process

For an overview of the search plan, articles published between the years 2010 to 2018 were included in the search (Fig. 1). The search was undertaken in May 2018 with the total number of articles found in the databases adding up to 38,559. A total of 37,850 articles were found in Google Scholar and 709 retrieved from PubMed. Careful reading of the titles of each article from both databases enabled researchers remove duplicates as well as include only articles on *P. falciparum*. This trimmed down the number of articles to 128 (Fig. 1). The Abstract of all 128 articles were read and articles that reported RDTs results, performance, presentation, interpretation and limitation were included for further review. This process reduced the articles to 74. Further review of the selected articles resulted in the exclusion of articles published before 2010, main text written in foreign languages and study location outside *P. falciparum* endemic areas. A total of 27 articles were finally selected for the review.

### Results

#### Interpreting RDT results

One of the challenges of the RDT, is the interpretation of the results, especially when compared with microscopy [10, 13]. There are instances where RDT will be positive but no parasites will be seen on microscopy, conversely, there are instances where RDT will be negative but microscopy will detect parasites in the blood [14]. There are instances too, when RDT will be positive but there is no clinical malaria or, the fever is not caused by malaria [15]. Despite the fact that RDTs have been recommended as a means of laboratory confirmation of malaria before the prescription of antimalarial, the interpretation of test results should be done with caution to ensure better clinical outcome in patient management [12–14].

#### RDT positive in a patient with fever

**Fig. 2:**
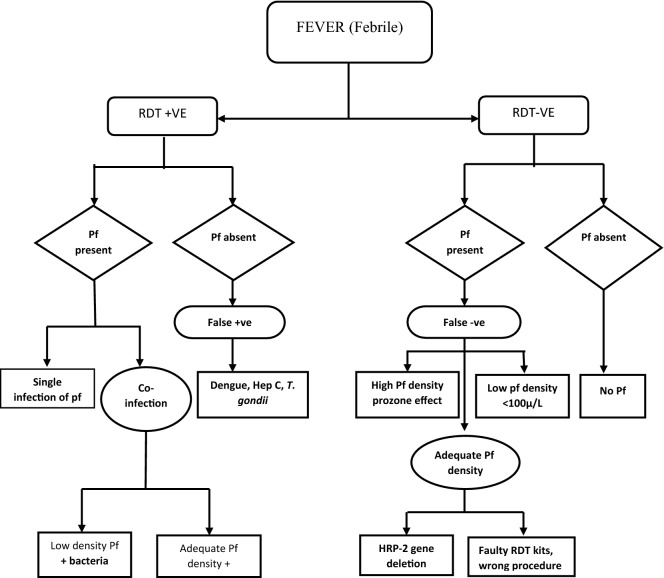
Possible presentation and interpretations of RDT result in a febrile patient

Malaria RDT positive results in patient with fever, aside malaria, can be caused by other possible infections (Fig. 2). It may be a risky clinical practice to solely consider malaria and ignore other possible causes of fever in a patient with positive RDT results. This is because it is possible that a patient with a positive RDT results might truly have *P. falciparum* infection but that infection may not be responsible for the fever (Fig. 2) [14]. This means that a patient can have *P. falciparum* infection but there will be no clinical malaria, suggesting that fever might be caused by another fever causing pathogen like bacteria or virus. This clinical scenario is not difficult to come by since asymptomatic *P. falciparum* infection with low parasite density is a common presentation in endemic areas [14, 15] and they can co-exist with several bacterial infections (Fig. 2) [14]. Therefore, relying solely on RDT positive results and subsequently treating for malaria may sometimes prove fatal for the patients [14].

There is also another possibility where the fever is indeed caused by *P. falciparum* infection together with a bacteria, making it a malaria and bacteria co-infection, which is another common presentation in malaria endemic area (Fig. 2) [16–18]. False positive in a patient with fever is also a possibility [19] and have been reported in patients with hepatitis C, dengue virus and *Toxoplasma gondii* infections (Fig. 2) [19, 20]. Immunologic cross-reaction from heterophile antigens produced by these pathogens have been implicated as the cause of these false positive results [21].

#### RDT negative in a patient with fever

Antimalarial treatment is not recommended in RDT negative patient with fever, as per the current malaria treatment protocol, such fevers are considered not to be caused by malaria. However, cases of false negative have been reported in patient with high parasite densities, a phenomenon called the prozone effect (Fig. 2) [12]. The prozone effect, also called the high dose-hook phenomenon is caused by excess parasite antigens binding with antigen detecting antibody with no epitope available for capture or test band antibody, to produce the test band result [12].

Genetic variation of *P. falciparum* HRP2 have also been implicated in some cases of false negative RDT (Fig. 2) [22, 23]. Reports show that HRP2 gene deletion in endemic areas, lead to absence of HRP2 antigens causing negative result with RDT [24]. However WHO, making a pronouncement on the issue of HRP2 genetic causing false negative RDT result, stated that HRP2 gene deletion might not be the main cause of false negative but rather malfunctioning RDTs due to poor storage and transport condition, operator errors and poor quality RDTs [25].

False negative can also occur at low parasite densities defined as < 100 asexual parasites/micro liter of blood or < 0.002% of red blood cells infected (Fig. 2) [12]. It is argued that low parasitaemia that is missed by RDT is usually of no clinical significant especially in adult [14]. However, care should be taken in infants and young children with fever and negative RDTs because their rudimentary immunity can allow low parasite density (undetected by RDT) to cause fever (Fig. 2) [12]. However, studies have shown that it is safe to withhold anti malaria from febrile infants and young children with negative RDT [26, 27].

RDT negative result in a patient with fever, have given rise to another nagging problem of over prescription of antibiotics [28, 29]. The main explanation for this is that the health worker assumes that a major non malaria cause of fever is probably a bacterial infection [29]. The interpretation and subsequent decision to give antibiotics to a febrile patient with negative RDT, might not be entirely wrong [29]. However, it is also possible that these patients might have only needed antipyretics [30] instead of antibiotics since some fevers can be caused by viruses [31, 32].

#### RDT positive in a patient without fever

**Fig. 3:**
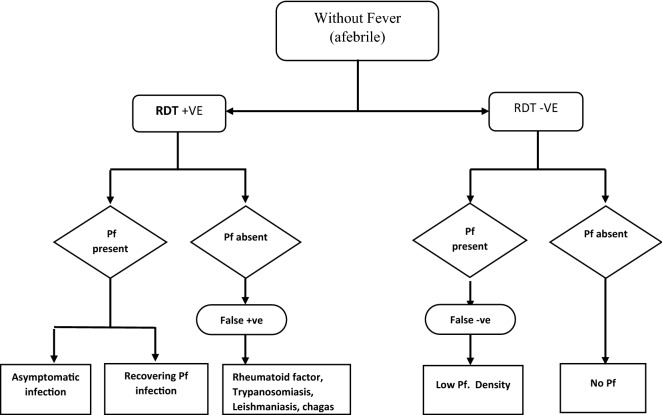
Possible presentation and interpretations of RDT result in an afebrile patient. RDT +VE: rapid diagnostic test is positive; RDT −VE: rapid diagnostic test is negative; Pf: *Plasmodium falciparum*

An afebrile patient testing positive for *P. falciparum* with RDT probably has an asymptomatic infection (Fig. 3). The preponderance of asymptomatic infections especially in older children and adults in endemic areas is a well-established fact [15]. However, classifying their status as heathy or unhealthy and if they require antimalarial treatment is where some argument still abound. Some studies show that asymptomatic infection confers protection and treating it can increase the risk of subsequent clinical malaria [33, 34]. Others did not show protection and also revealed that treatment did no increase the risk of subsequent clinic malaria [35, 36]. Some studies even show that asymptomatic infection is detrimental to the health and intellectual development of children [14, 37].

Positive RDT results without fever can also occur in patients recovering from malaria after treatment (Fig. 3) [38]. This is because despite successful parasite clearance there can be persistent circulating HRP2 antigens in the blood [38, 39]. These antigens can persists weeks after treatment resulting in positive result, wrong diagnosis and unnecessary treatment of malaria, defeating the whole aim of RDT which is to prevent misdiagnosis and treatment [38, 40].

In some situations, there might not be any parasite or antigens at all in the person’s blood stream and RDT will still be positive. Certain non-fever causing diseases can produce heterophile antigens that can react with test kits antibodies to produce a false positive results [19]. False positive results can arise from disease like Chagas diseases, leshmaniasis, trypanosomiasis and patients with rheumatoid factor (Fig. 3) [19–21].

#### RDT negative in a patient without fever

A patient without fever and testing negative for malaria with RDT is most likely free from malaria. However, if they are asymptomatic, their true status will evade RDT especially if the parasite density is very low (Fig. 3) [12]. It will be appropriate not to treat patient with negative RDT especially when afebrile. But again, cases have been reported where health workers treat patients with anti malarials, although they tested negative for RDT and are afebrile, thereby showing a total disregard of the negative RDT result [19].

### Conclusion

The introduction of RDTs have reduced over prescription and indiscriminate use of antimalarial in endemic areas. However, RDT may be flawed by some weakness in estimating parasite densities and inability to detect parasites at low densities. Furthermore, its inability to differentiate between the exact causes of fever in patients with malaria and bacterial infection or other fever causing pathogens has made it dicey in managing febrile patients with antimalarial alone. There is also the problem of false positive results from diseases that produce heterophile antigens and false negative from, prozone effect, HRP2 gene deletion and poor quality RDT.

These weaknesses in malaria RDT kits shows that it cannot solve all the problems of malaria diagnosis in endemic areas. The success of RDT cannot drown the nagging need of a scale up of laboratory facilities in resource limited settings. Expert microscopy should be the goal, together with other necessary laboratory tools to aid in the overall diagnosis of febrile and afebrile patients in malaria endemic areas.

### Limitations of the study

Two search engines were used for this review and the researchers acknowledge that it limited the number of studies identified with the subject of interest. This notwithstanding, the articles retrieved present a valid reasoning on the need to be circumspect in the interpretation of RDT test results in endemic regions.

## Abbreviations

WHO: World Health Organization
IMCI: Integrated Management of Childhood Illness
RDT: Rapid Diagnostic Test
SD: Standard Diagnostics
HRP2: Histidine Rich Proteins-2 (HRP2)
pLDH: Parasite Lactose Dehydrogenase Enzyme

## References

[1] Febir LG, Baiden FE, Agula J, Delimini RK, Akpalu B, Tivura M (2015). Implementation of the integrated management of childhood illness with parasitological diagnosis of malaria in rural Ghana: health worker perceptions. Malar J..

[2] World Health Organization. \tWHO: guideline for the treatment of malaria. 2nd ed. Geneva: World Health Organization; 2010. Nature. 2010;284. http://www.who.int.

[3] Gove S (1997). Integrated management of childhood illness by outpatient health workers: technical basis and overview. Bull World Health Organ.

[4] Azikiwe CCA, Ifezulike CC, Siminialayi IM, Amazu LU, Enye JC, Nwakwunite OE (2012). A comparative laboratory diagnosis of malaria: microscopy versus rapid diagnostic test kits. Asian Pac J Trop Biomed..

[5] Moody A (2002). Rapid diagnostic tests for malaria parasites. Clin Microbiol Rev.

[6] Chandler CI, Whitty CJM, Ansah EK (2010). How can malaria rapid diagnostic tests achieve their potential? A qualitative study of a trial at health facilities in Ghana. Malar J..

[7] World Health Organization (2015). Guidelines for the treatment of malaria.

[8] Wongsrichanalai C, Barcus MJ, Muth S, Sutamihardja A, Wernsdorfer WH (2007). A review of malaria diagnostic tools: microscopy and rapid diagnostic test (RDT). Am J Trop Med Hyg.

[9] Wilson ML (2012). Malaria rapid diagnostic tests. Clin Infect Dis..

[10] Dozie U, Chukwuocha U. Comparative evaluation of malaria rapid diagnostic test kits commercially available in parts of south eastern Nigeria. J Trop Dis. 2016;04(02). 10.4172/2329-891X.1000201, http://www.esciencecentral.org/journals/comparative-evaluation-of-malaria-rapid-diagnostic-test-kits-commercially-available-in-parts-of-south-eastern-nigeria-2329-891X-1000201.php?aid=69768.

[11] World Health Organization. Malaria RDTs—mechanism of action—WHO Western Pacific Region; 2005.

[12] Gillet P, Mori M, Van Esbroeck M, Van Den Ende J, Jacobs J (2009). Assessment of the prozone effect in malaria rapid diagnostic tests. Malar J..

[13] Abdulkadir I, Rufai HA, Ochapa SO, Malam MS, Garba BI, Oloko AGY (2015). Malaria rapid diagnostic test in children: the Zamfara, Nigeria experience. Niger Med J..

[14] Bisoffi Z, Gobbi F, Buonfrate D, Van den Ende J (2012). Diagnosis of malaria infection with or without disease. Mediterranean J Hematol Infect Dis..

[15] Walldorf JA, Cohee LM, Coalson JE, Bauleni A, Nkanaunena K, Kapito-Tembo A (2015). School-age children are a reservoir of malaria infection in Malawi. PLoS ONE..

[16] Gómez-Pérez GP, Van Bruggen R, Grobusch MP, Dobaño C (2014). *Plasmodium falciparum* malaria and invasive bacterial co-infection in young African children: the dysfunctional spleen hypothesis. Malaria J..

[17] Church J, Maitland K (2014). Invasive bacterial co-infection in African children with *Plasmodium falciparum* malaria: a systematic review. BMC Med..

[18] Nielsen MV, Amemasor S, Agyekum A, Loag W, Marks F, Sarpong N (2015). Clinical indicators for bacterial co-infection in Ghanaian children with *P. falciparum* infection. PLoS One..

[19] Maltha J, Gillet P, Jacobs J (2013). Malaria rapid diagnostic tests in endemic settings. Clin Microbiol Infect..

[20] Lee J-H, Jang JW, Cho CH, Kim JY, Han ET, Yun SG (2014). False-positive results for rapid diagnostic tests for malaria in patients with rheumatoid factor. J Clin Microbiol..

[21] Haberichter KL, Johnson PC, Chittick PJ, Millward P, Robinson-Dunn B, Boyanton BL (2017). The brief case: false-positive rapid malaria antigen test result in a returned traveler. J Clin Microbiol..

[22] Okoth SA, Abdallah JF, Ceron N, Adhin MR, Chandrabose J, Krishnalall K (2015). Variation in *Plasmodium falciparum* histidine-rich protein 2 (Pfhrp2) and *Plasmodium falciparum* histidine-rich protein 3 (Pfhrp3) gene deletions in Guyana and Suriname. PLoS ONE..

[23] Muzamil AH, AwadElgeid M, Nasr A (2017). Gene variation and suspected *Plasmodium falciparum* histidine-rich protein 2 gene deletion and its impact on sensitivity of malaria rapid diagnostic testing in Sudan. BMJ Global Health..

[24] Amir A, Cheong F-W, De Silva JR, Lau Y-L (2018). Diagnostic tools in childhood malaria. Parasit Vectors..

[25] World Health Organization (2017). False-negative RDT results and implications of new reports of *P. falciparum* histidine-rich protein 2/3 gene deletions.

[26] Acremont V, Malila A, Swai N, Tillya R, Kahama-Maro J, Lengeler C (2010). Withholding antimalarials in febrile children who have a negative result for a rapid diagnostic test. Clin Infect Dis..

[27] Ka K, Bjo A, Mubi M, Janson A, Warsame M, Mårtensson A (2011). Malaria rapid testing by community health workers is effective and safe for targeting malaria treatment: randomised cross-over trial in Tanzania. PLoS ONE..

[28] Baiden F, Webster J, Owusu-Agyei S, Chandramohan D (2011). Would rational use of antibiotics be compromised in the era of test-based management of malaria?. Trop Med Int Health.

[29] Hopkins H, Hopkins H, Bruxvoort KJ, Cairns ME, Chandler CI, Leurent B (2017). Impact of introduction of rapid diagnostic tests for malaria on antibiotic prescribing: analysis of observational and randomised studies in public and private healthcare settings. BMJ..

[30] Roberts L (2017). Revolutionary malaria tests have unexpected downsides. Science..

[31] Lukšić I, Kearns PK, Scott F, Rudan I, Campbell H, Nair H (2013). Viral etiology of hospitalized acute lower respiratory infections in children under 5 years of age—a systematic review and meta-analysis. Croat Med J..

[32] Asante KP, Owusu-Agyei S, Cairns M, Boamah E, Manu G, Twumasi M (2016). Non-malaria fevers in a high malaria endemic area of Ghana. BMC Infect Dis..

[33] Liljander A, Bejon P, Mwacharo J, Kai O, Ogada E, Peshu N (2011). Clearance of asymptomatic *P. falciparum* infections interacts with the number of clones to predict the risk of subsequent malaria in Kenyan children. PLoS ONE..

[34] Sonden K, Doumbo S, Hammar U, Vafa Homann M, Ongoiba A, Traord B (2015). Asymptomatic multiclonal *Plasmodium falciparum* infections carried through the dry season predict protection against subsequent clinical malaria. J Infect Dis.

[35] Portugal S, Tran TM, Ongoiba A, Bathily A, Li S, Doumbo S (2017). Treatment of chronic asymptomatic *Plasmodium falciparum* infection does not increase the risk of clinical malaria upon reinfection. Clin Infect Dis.

[36] Buchwald AG, Sixpence A, Chimenya M, Damson M, Sorkin JD, Wilson ML (2018). Clinical implications of asymptomatic *Plasmodium falciparum* infections in Malawi. Clin Infect Dis..

[37] Vorasan N, Pan-Ngum W, Jittamala P, Maneeboonyang W, Rukmanee P, Lawpoolsri S (2015). Long-term impact of childhood malaria infection on school performance among school children in a malaria endemic area along the Thai–Myanmar border. Malar J..

[38] Grandesso F, Nabasumba C, Nyehangane D, Page A-L, Bastard M, De Smet M (2016). Performance and time to become negative after treatment of three malaria rapid diagnostic tests in low and high malaria transmission settings. Malar J..

[39] Kilauzi AL, Mulumba JGT, Magafu MGMD, Matchaba-Hove R, Tapera R, Magafu NS (2015). SD Bioline malaria antigen Pf (HRP-2/pLHD) for assessing efficacy of artemisinin combination therapy against *Plasmodium falciparum* in pediatric patients in the democratic republic of the Congo. Pan Afr Med J..

[40] Kiemde F, Bonko MDA, Tahita MC, Lompo P, Rouamba T, Tinto H (2017). Accuracy of a *Plasmodium falciparum* specific histidine-rich protein 2 rapid diagnostic test in the context of the presence of non-malaria fevers, prior anti-malarial use and seasonal malaria transmission. Malar J..

